# The Medical Needs of the Army

**Published:** 1915-04-03

**Authors:** 


### The Hospital

The Workers' Newspaper of Administrative Medicine and Institutional Life, Administration, National Insurance and Health.

No. 1502, Vol. LVIII. SATURDAY, APRIL 3, 1915. PRICE ONE PENNY.

230054

### THE MEDICAL NEEDS OF THE ARMY.

The call of the Army authorities for increased help from the medical profession is an event of such acute importance that we need make no apology for again returning to the subject. It concerns the protection of interests which are just now peculiarly the subject of national regard, and it raises the large and special responsibility which the profession owes to these interests. Unless the claim is met promptly and adequately members of the profession will not readily find a plea which will command the general judgment of their fellow-countrymen. Munitions, men, and doctors are demanded with almost equal insistence, and failure in either of these directions will surely not escape censure. If adequate supplies of the first two are not forthcoming there have been fairly plain hints that compulsion will be applied. It would be pitiable that the same discipline should appear as an official threat to the medical profession. None the less is it true that unless more doctors are engaged in the service of the Army the defect will create an acute national emergency. This is, in brief, the position to which the profession must address itself, and the country will expect that the responsibility is fully recognised and efficiently discharged.

The appeal issued by the Director-General was made public in the middle of March, and it is therefore now opportune to inquire what reply has been made to it. No one outside the official secrets of the War Office can, of course, pretend to say what offers may have been made by individuals, but so far little evidence of activity has been betrayed by the corporate organisations of the profession. Yet surely upon these there weighs the plain duty of guidance and direction. No one can doubt the existence among their members of abundant patriotism and good will, but there is needed skill and authority to organise these qualities for practical ends. The necessary machinery for this end exists, and it is the duty of those who can control it to set it in operation. For this there is the more need, as evidences are not wanting of the risk of some misunderstanding between the interests immediately concerned. There is some measure of uncertainty both as to what exactly the War Office wishes, and what the profession is able to give in view of its admitted and important responsibilities to the civil population. In these circumstances the plain need is for a conference between representatives authorised to express the views of those for whom they speak and competent to give guarantees both on one side and on the other. The sole desire of the profession is to render help, but this must be reconciled with the equal claims of the general community. Both ambitions are possible provided only that the work in each department is wisely and scientifically organised.

We may allude briefly to one or two of the difficulties of the situation. First, it is beyond question that the Director-General's letter came as a great surprise to the profession. The current belief had been that the War Office had more medical volunteers than it required, and it has been stated openly within the last few days that hospital physicians and surgeons of undoubted competence and status who volunteered at the very beginning of the war have never received any response from the War Office other than a formal acknowledgment of their offer. It can hardly be a matter for surprise that some difficulty is experienced in harmonising such a statement with the acute note of the Director-General's letter. No doubt there is some explanation of the discrepancy, but until it is afforded, conviction of the full measure of the emergency is hardly likely to be secured.

In the second place, it is pointed out that the official request is that "every man who can arrange for his work to be done at home" should at once come forward, which of course means that he should free himself from his ordinary obligations to the civil community. But the numbers of those who are free to take this step are now inconsiderable, seeing that the great majority of them are already engaged in military service. Already the civil hospitals are carrying on their work under great difficulties in consequence of the number of

HO69X

members of the visiting staffs who have joined the Army, and qualified juniors for the resident staffs are not to be had on any terms. In many places, too, the strain of private practice has greatly increased owing to the absence of many practitioners in response to the claims of the combatant services. One evidence of this is the appeal addressed to the younger teachers in the medical schools to sacrifice the holiday which usually comes to them at the end of the winter session in order to afford a respite to overworked country practitioners. There are, indeed, few practitioners left who are able to "arrange for their work to be done at home," for the simple reason that the great majority who held positions admitting such an arrangement are already working with the naval and military Forces. In its present form, therefore, the Director-General's appeal is likely, it is to be feared, to meet with but a scanty response.

Yet it is certain that there are many doctors willing and anxious to help. They can give some of their time, though not without sacrifice, but it is impossible for them to neglect the obligations they have undertaken to hospital and private patients. According to some, such partial service is not acceptable to the authorities, and certainly it has not been asked for. Naturally the desire is to have doctors as a part of the military machine, but certain conspicuous exceptions to this have already been made, and the process, it seems to us, will have to be extended. The essential claim is for an adjustment between those who want help and those who can render it. Each side must recognise the difficulties of the position, and with a little frank conversation these would all, we are convinced, be readily surmounted. But until the policy of the round table is adopted progress will be difficult to secure.

